# Lyoniresinol 3α-O-β-D-Glucopyranoside-Mediated Hypoglycaemia and Its Influence on Apoptosis-Regulatory Protein Expression in the Injured Kidneys of Streptozotocin-Induced Mice

**DOI:** 10.1371/journal.pone.0081772

**Published:** 2013-12-03

**Authors:** Qingwei Wen, Tao Liang, Feizhang Qin, Jinbin Wei, Qiaoling He, Xiu Luo, Xiaoyu Chen, Ni Zheng, Renbin Huang

**Affiliations:** Pharmaceutical College, Guangxi Medical University, Nanning, Guangxi, China

## Abstract

*Averrhoa carambola* L. (Oxalidaceae) root (ACLR) has a long history of use in traditional Chinese medicine for treating diabetes and diabetic nephropathy (DN). (*±*)-*Lyoniresinol 3α-O-β-D-glucopyranoside* (LGP1, LGP2) were two chiral lignan glucosides that were isolated from the ACLR. The purpose of this study was to investigate the effect of LGP1 and LGP2-mediated hypoglycaemia on renal injury in streptozotocin (STZ)-induced diabetic mice. STZ-induced diabetic mice were administrated LGP1 and LGP2 orally (20, 40, 80 mg/kg body weight/d) for 14 days. Hyperglycaemia and the expression of related proteins such as nuclear factor-κB (NF-κB), caspase-3, -8, -9, and Bcl-associated X protein (Bax) were markedly decreased by LGP1 treatment. However, LGP2 treatment had no hypoglycaemic activity. Diabetes-dependent alterations in the kidney such as glomerular hypertrophy, excessive extracellular matrix amassing, and glomerular and tubular basement membrane thickening were improved after 14 days of LGP1 treatment. B cell lymphoma Leukaemia-2 (Bcl-2) expression was reduced in the STZ-induced diabetic mouse kidneys but was enhanced by LGP1 treatment. These findings suggest that LGP1 treatment may inhibit diabetic nephropathy progression and may regulate several pharmacological targets for treating or preventing diabetic nephropathy.

## Introduction

Diabetes mellitus (DM) is a metabolic disease that is characterized by chronic hyperglycaemia, which is caused by islet β-cell dysfunction and peripheral tissue insulin resistance (IR). DM has become a global disease that absorbs major public health resources and bears a heavy burden in both industrialized and developing countries[1]. DM-induced morbidity, disability, lethality rate and bodily damage is in 3rd place among non-communicable diseases. According to the International Diabetes Federation (IDF), worldwide DM prevalence was 366 million in 2011, and it is projected that DM prevalence will reach 552 million by 2030[2,3]. Asia emerged as an epicentre of the diabetes epidemic at the turn of the 20th century. Presently, DM incidence in China is approximately 40 million people; of these, more than 90% have type 2 diabetes mellitus (T2DM), and over 80% suffer from obesity[4]. Target tissues such as skeletal muscle, liver and adipose tissue display insulin insensitivity, which is a primary, independent risk factor for T2DM[5]. Once the β-cell compensatory ability has been attenuated, the body would be chronically hyperglycaemic, which would cause T2DM. Increasing evidence has revealed a clear association among obesity, inflammation and IR[6]. In human IR states, the inflammatory marker C-reactive protein (CRP) is commonly elevated, and NF-κB plays a key role in inflammation and IR[7].

The NF-κB/Rel family includes NF-κB1 (p50/p105), NF-κB2 (p52/p100), p65 (RelA), RelB, and c-Rel. NF-B hetero- or homodimers such as p50/p65, p52/p65, and p50/50 are commonly associated with regulatory inhibitors of κB (IκB) proteins, of which the most important may be IκBα, IκBβ, and IκBε, which exist as inactive cytoplasmic proteins[8]. These proteins are activated by stimuli such as mucopolysaccharide from bacteria, viruses, oxyradicals and many cytokines. The active NF-κB triggers the transcription of target genes TNF-*α*, IL-1β, IL-6, NOS, MCP-1, VCAM-1, ICAM-1, ECAM-1 to increase their gene expression to promote inflammation[9,10]. These proinflammatory mediators then act as new stimuli to further activate NF-κB and induce the corresponding gene transcription, which results in more low-grade inflammation and a vicious circle. Moreover, these inflammatory cytokines activate serine kinases in the insulin signalling pathway to phosphorylate threonine and serine residues on insulin receptor substrate (IRS) and PI3K pathway proteins, which inhibits insulin-stimulated IRS and PI3K tyrosine phosphorylation and blocks insulin signal transduction leading to IR[11,12].

Approximately 40 percent of type 2 diabetic patients develop in DN [13]. DN is characterized by excessive extracellular matrix with glomerular and tubular basement membrane thickening, which ultimately progresses to glomerulosclerosis and tubulo- interstitial fibrosis. DN is a common and serious microangiopathy complication that has become the leading cause of end-stage renal disease. Apoptosis was approximately 6- and 3-fold higher in the glomeruli and renal tubules of early nephropathy patients compared with normal persons, respectively[14]. Many publications indicated that hyperglycaemia, oxidative stress and caspase family members played a key role in the cell apoptosis, which correlated to islet β-cell death and DN. Caspase family members amino acid sequences, structures and enzyme properties are usually similar; they exist in procaspase form and are inactive in cells. To date, there are at least three ways to activate the procaspase, which includes autoactivation, transactivation, and noncaspase proteinase activation. Once apoptosis is triggered, the cascade is amplified. Caspase family members are divided into two types. The initiator caspases including caspase-2, 8, 9, 10 are upstream of the caspase cascade and can autoactivate and activate downstream caspases in concert with other protein factors. The executioner caspases include caspase-3, 6, and 7, which cleave their substrates and alter cellular biochemical characteristics and morphology, eventually causing apoptosis[15,16]. Caspase-3 is a crucial protease that promotes cell death once activated, and the downstream apoptotic cascade would be inevitably triggered. Therefore, caspase-3 is known as the “death protease”[17].

Apoptosis can be controlled by apoptosis-regulating proteins in cells. Bcl-2 and Bax family members are apoptosis-regulating proteins that play a key role in apoptosis caused by various stimuli. Bcl-2 and Bax directly related to apoptosis regulation. Increased Bax expression promotes apoptosis while increased Bcl-2 expression inhibits it[18].

ACLR is a perennial herb that is widely distributed in China, Taiwan, Malaysia, India, Brazil, and America. Its root has been used for thousands of years in traditional Chinese medicine to remedy lithangiuria, arthralgia, and chronic paroxysmal headache. Our previous studies and other publications have reported the isolation and identification of some compounds such as β-sitosterol, lupeol, 1,5-dihydroxy-6,7-dimethoxy-2-methyl- anthraquinone 3-*O*-*β*-glucopyranoside, 3,4,5-trimethoxyphenol-1-*O*-*β*-*D*-glucopyranoside, benzyl-1-O-β-D-glucopyranoside, (+)-5'-methoxyisolariciresinol 3α-*O-β*-*D*-gluco- pyranoside, and (+)-isolariciresinol 3α-*O-β*-*D*-glucopyranoside[19,20]. ACLR leaf and root extracts have also demonstrated hypoglycaemic, hypotriglyceridemic, anti-lipid peroxidative and anti-atherogenic effects in STZ-induced diabetic rats (or mice)[21,22].

To provide insight into the anti-diabetic and hypoglycaemic mechanisms of LGP1 and LGP2, ACLR extract in 60% aqueous ethanol (aq. EtOH) was suspended in H_2_O and further extracted with cyclohexane, ethyl acetate (EtOAc) and *n*-butanol (BuOH). The BuOH extract was successively purified by open repeated silica gel, Sephadex LH-20, ODS column and P-HPLC to obtain LGP1 and LGP2. These compounds were administrated to STZ-induced mice for 14 days with pioglitazone (Pio.) as a positive control to evaluate its hypoglycaemic effect and its anti-apoptotic mechanism, which was relevant to caspase-3, 8, 9, NF-κB, Bcl-2 and Bax.

## Materials and Methods

### 2.1: General

Melting points were measured without correction on a binocular microscopic X-5 melting point apparatus (Beijing, China). The FTIR data were recorded on a PekinElmer FTIR spectrophotometer (Spectrum One) with a DGTS detector. ^1^H and ^13^C- NMR spectra data were obtained in MeoD and C_5_D_5_N on a Bruker Av 600 instrument at 600 and 150 MHz, respectively. Chemical shifts were expressed in δ (ppm) with TMS as an internal standard. The P-HPLC was run on a Shimadzu LC-8A that had been equipped with a SPD-10A VP detector and an AQ-C18 column (20×250 mm, 10 μm). Open column chromatography (CC) was performed using silica gel (200-300 mesh, Qingdao Marine Chemical Ltd.), ODS-AQ-HG (YMG*GEL, 12 nm, S-50 μm, Lot: 9955), and Sephadex LH-20 (20-100 μm, GE Healthcare). TLC was performed on silica gel plates (Qingdao Marine Chemical Ltd., Qingdao, China). Chromatographic grade methanol was used, and all of the other reagents were of analytical grade and purchased from the Tanjin Damao Chemical Reagent Factory (Tanjin, China).

Fasting blood glucose (FBG) and insulin concentrations were measured with the Roche ACCU-CHEK^®^ Performa (Strip lot: 470664, Switzerland) and an Iodine [^125^I] Insulin Radioimmunoassay Kit (Lot: 120520, Beijing North Institute of Biological Technology, Beijing, China) with a DFM-96 Zhongcheng gamma counter (Hefei, China). An in situ cell death detection kit,pod (Lot: 12058700, Germany) and DAB substrate chromogenic reagent (Lot: 201201, Shanghai, China) were purchased from Roche Diagnostics GmbH, Shanghai Long Island Biotec. Co., LTD. 

All of the images were acquired in ten views that were selected randomly in each section from each animal by light microscopy (CX31, Olympus, Japan) at 400× magnification, and the Integrated optical density (IOD) value and apoptotic cell content were calculated with Image-Pro Plus 6.0 software (Media Cybernetics, USA).

### 2.2: Plant material

ACLR were collected from Linshan County, Guangxi Province, China, in June 2010 and were identified by Pro. Lai Mao-xiang. The collections were carried out on private land, and the owner of the land has confirmed and given permission for us to conduct the study on the site. The voucher specimen (No. 20100605) was deposited in the Guangxi Institute of Chinese Medicine &Pharmaceutical Science herbarium (Guangxi, China).

### 2.3: Antibodies

Caspase-3 (E87, Lot: 931675, abCam, London, U.K.), caspase-8 (mouse monoclonal IgG1, Lot#H2508, Santa Cruz Biotechnology, Inc., California, U.S.A.), caspase-9 (mouse mAb, Lot: 7, Cell Signaling Technology, Boston, U.S.A.), NF-kappa B p65 (E498, Lot: 1, rabbit ab, Cell Signaling Technology, Boston, U.S.A.), Bcl-2 (mouse monoclonal IgG1, Lot#D0108, Santa Cruz Biotechnology, Inc., California, U.S.A.), Bax (rabbit polyclonal IgG, Lot#B1811, Santa Cruz Biotechnology, Inc., California, U.S.A.). Anti-mouse/rabbit (HRP, lot 201201, Shanghai Long Island Biotec. Co., LTD, Shanghai, China);

### 2.4: LGP isolation

**Figure 1:**
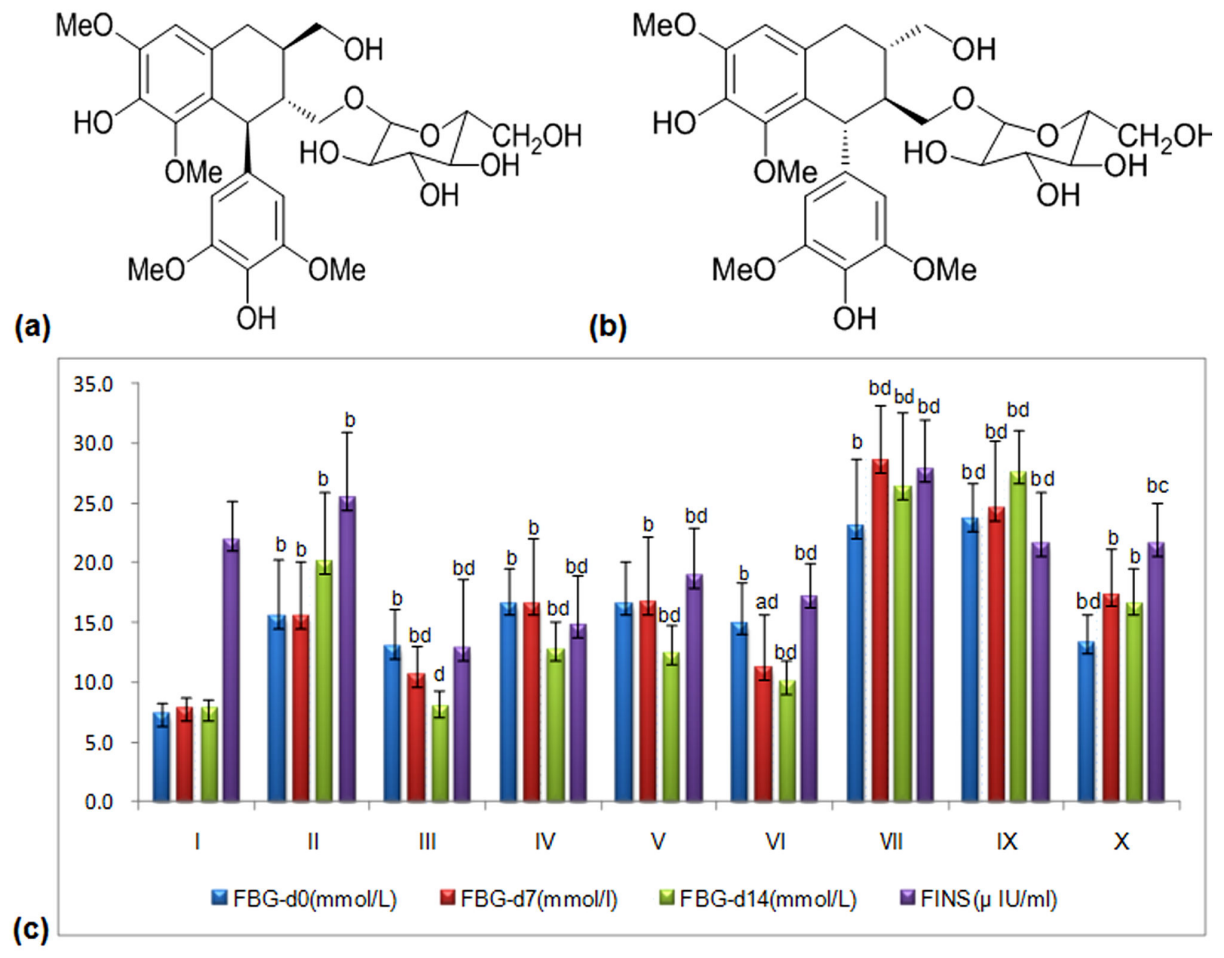
(a) Structure of (+)-*Lyoniresinol 3α-O-β-D-glucopyranoside* (LGP1); (**b**) Structure of (-)-*Lyoniresinol 3α-O-β-D-glucopyranoside* (LGP2). (**c**) Hypoglycemic effect induced by LGP1 and LGP2 treatments. i: Normal control; ii: Model control; iii: 10 mg.kg^-1^.d^-1^ of Pioglitazone; iv-vi: 20, 40, 80 mg.kg^-1^.d^-1^ of LGP1; vii-ix: 20, 40, 80 mg.kg^-1^.d^-1^ of LGP2. Results were presented as the means ±SD. ***^a^*** *P*<0.05 and ^b^ *P*<0.01 as compared with normal control group; ^c^ *P*<0.05 and ^d^ *P*<0.01 as compared with model group.

The air-dried and powdered ACLR (12kg). was extracted with 60% aq. EtOH (3×96 L, 1 h each). The ethanolic solution was concentrated under a vacuum to yield a syrup-like extract (20 L), which was suspended in H_2_O and extracted with cyclohexane (3×20 L), EtOAc (3×20 L), and n-BuOH (3×20 L). The n-BuOH extract (153 g) was separated with repeat silica gel column chromatography (open column 13×100 cm, 200–300 mesh, 1.5 kg) that was eluted successively with gradient petroleum ether/ EtOAc (100:0, 1:1, 0:100) and EtOAc/methanol (18:0, 10:1, 8:1, 6:1, 4:1, 2:1, 0:100). The fractions were then purified using Sephadex LH-20 (2.0×60 cm, methanol) and RP-18 (ODS-AQ-HG, 5.0×60 cm, 12 nm S-50 μm) columns and P-HPLC (methanol/H2O 28:72, 8.0 mL/min, 203 nm) to obtain two lignan chiral glycosides: (*+*)*-Lyoniresinol 3α-O-β-D-glucopyranoside* (418.9 mg, **LGP1**) and (*-*)*-Lyoniresinol 3α-O-β-D-glucopyranoside* (534.7 mg, **LGP2**)[20]. The structures of LGP1 and LGP2 are illustrated in Figure **1(a-b)**, respectively.

### 2.5: Animals and treatments

Kunming mice of either sex weighing 18-22 g from the Experimental Animal Centre of Guangxi Medical University (registration number SCXK 2009-0002) were used for the study. The mice were housed in plastic cages with controllable temperature and humidity under a 12 h light/dark cycle and were fed a standard diet and water ad libitum. All of the animal experimental procedures and protocols were approved by the institutional ethical committee of Guangxi Medical University (approval No. 2012011121).

To establish STZ-induced diabetes, the mice were fasted overnight and treated with freshly-prepared STZ that had been dissolved in saline at 120 mg/kg body weight via tail vein injection[23,24]. Diabetes was confirmed by fasting blood glucose level determination (FBG ≥ 11.1 mmol/L) on the third day post-STZ administration. The animals were randomly divided into 9 groups (n =10 mice per group) as follows: groups 1 and 2 were normal and T2DM mice (control), which were both given saline (vehicle); group 3 was diabetic and was treated intragastrically with 10 mg.kg^-1^.d^-1^ Pio.; groups 4-6 and 7-9 were diabetic and treated intragastrically with 20, 40, 80 mg.kg^-1^.d^-1^ of LGP1 or LGP2, respectively.

After 14 days, blood was collected from the intraocular canthal, and the mice were sacrificed by cervical dislocation. The blood was centrifuged at 3000 rpm for 15 minutes, and the serum was transferred into new tubes that were stored at -20 celsius degree until further analysis. Their kidneys were harvested, immediately fixed in 10% formaldehyde solution, embedded in paraffin and sectioned at 5 μm for further analysis.

### 2.6: FBG assay

During the experiment, fasting blood glucose was measured from the tail vein on d0, d7, and d14 according to the Roche ACCU-CHEK^®^ Performa operation instructions, and the following formula to calculate the FBG descent rate (%).

### 2.7: Fasting insulin (FINS) and Insulin sensitivity index (ISI)

Serum was thawed at room temperature, and serum insulin levels were quantified using a commercial iodine [^125^I] insulin radioimmunoassay kit. Each assay was performed following the kit instructions. A series of standard concentrations were assayed in parallel with the samples. The sample insulin concentrations were calculated using the corresponding standard curves and expressed as mU/L.

ISI was calculated according to the fasting insulin and glucose concentration. The ISI formula is given below[25].

### 2.8: Histopathological examination

Paraffin-embedded slides were dewaxed, rehydrated and H&E stained according to standard pathologic procedures. In brief, slides were deparaffined in dimethylbenzene, hydrated in gradient ethanol, washed with distilled water, and hematine-eosin (HE) stained, in which the nuclei were blue and the cytoplasm was red, and the cells were then imaged using a 400× light microscope. In addition, kidney injuries were evaluated and represented by the method of tubular necrosis-individual severity index (TN-ISI) score as described previously[26].

### 2.9: Apoptosis evaluation

Cell apoptosis was analysed by terminal deoxynucleotidyl transferase (TdT)-mediated deoxyuridine triphosphate (dUTP) nick end labelling (TUNEL). Briefly, according to the in situ cell death detection kit, POD (Lot: 12058700, Roche Diagnostics GmbH, Germany) instructions, the slides were dewaxed, rehydrated and washed with 0.01 M PBS (pH 7.4, 3 min×3), incubated with proteinase K (10~20 μg/ml, dissolved with 10 mM Tris/HCl, pH 7.4~8) for 30 min at 37°C, and rinsed 3 times with PBS. The sections were then incubated with TUNEL reaction mixture for 1h at 37°C and rinsed 3 times with PBS. The converter-POD was added and incubated for 30 min at 37°C, followed by washing 3 times with PBS. The sections were then stained with DAB substrate and haematoxylin, dehydrated by gradient alcohol, made transparent in dimethylbenzene and sealed with neutral rubber[27]. Cells with brown nuclear granulation were considered to be positive. Using a 400× light microscope, ten sections were randomly chosen from each experimental mouse and observed in ten slide views.

### 2.10: Immunohistochemical analysis

All of the procedures were strictly performed in accordance with the manufacturer’s directions. Briefly, paraffin-embedded slides were dewaxed, rehydrated and rinsed 3 times with PBS. Antigens were retrieved in boiling citrate buffer solution (0.01 mol/L, pH 6.0) for 90 s followed by 3 PBS washes. Slides were then coincubated with 3% H_2_O_2_ for 15 min, rinsed 3 times with PBS and blocked with non-immune sera for 40 min. The slides were coincubated with primary antibodies [caspase-3, 8, 9 (1:200); NF-κB p65, Bcl-2, Bax (1:400)] overnight at 4°C, rinsed 3 times with PBS, incubated with anti-mouse/rabbit (HRP) antibodies (1:400) for 20 min, stained with DAB substrate chromogenic reagent and hematoxy, dehydrated, and imaged by light microscopy (CX31, Olympus, Japan) at 400× magnification[28]. 

### 2.11: Statistical analysis

The results are presented as the mean *±* standard deviation ( x ¯±s) . The statistical analyses were performed using SPSS 19.0 software (Chicago, IL, USA). Statistical significance between the groups was assessed by one-way analysis of variance (ANOVA) followed by student’s test. For all analyses, *p*≤0.05 was considered to be statistically significant.

## Results

### 3.1: FBG, FINS and ISI serum levels

**Figure 2:**
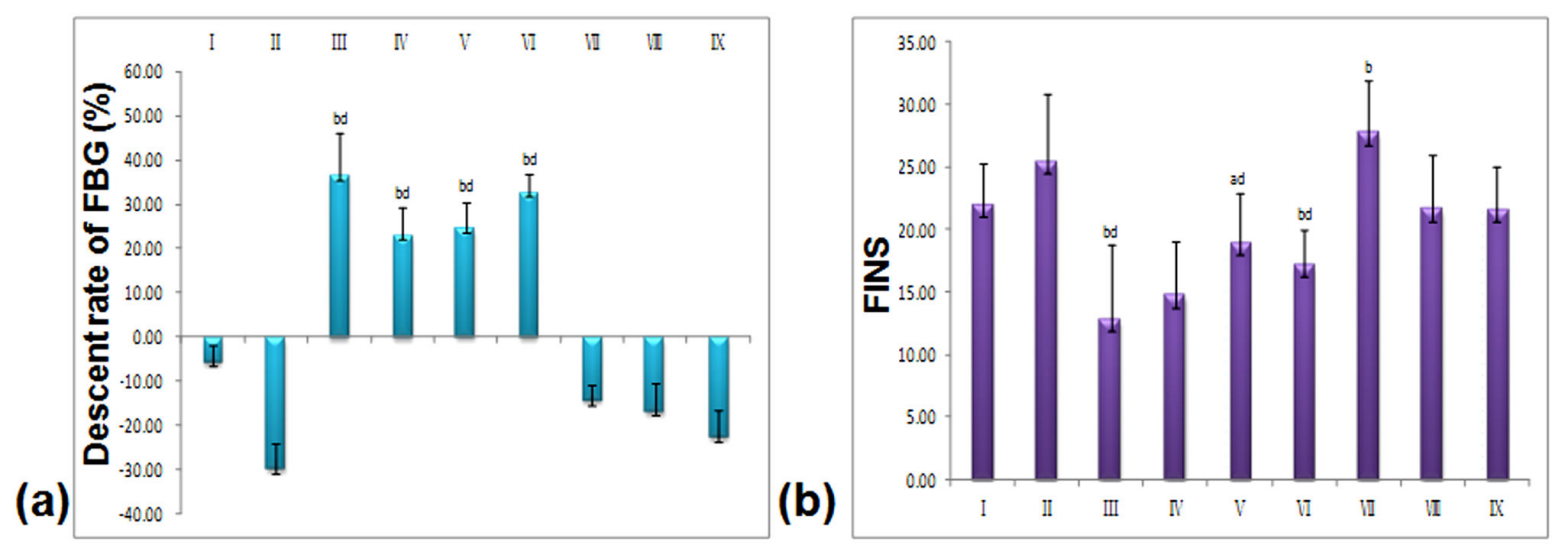
(a) Descent rate of FBG (%) induced by LGP1 and LGP2 treatments. (**b**) Fasting Insulin (FINS). i: Normal control; ii: Model control; iii: 10 mg.kg^-1^.d^-1^ of Pioglitazone; iv-vi: 20, 40, 80 mg.kg^-1^.d^-1^ of LGP1; vii-ix: 20, 40, 80 mg.kg^-1^.d^-1^ of LGP2. The results were presented as the means ±SD. ***^a^*** *P*<0.05 and ^b^ *P*<0.01 as compared with normal control group; ^c^ *P*<0.05 and ^d^ *P*<0.01 as compared with model group.

As demonstrated in Figure **2a**, after 14 days, a significant decrease in serum FBG levels was observed in the low, medium and high dose LGP1-treated animals (12.8±2.4 mmol/L, 12.5±2.3 mmol/L, 10.0±1.8 mmol/L, respectively) compared with the model control (15.5±4.6 mmol/L). FBG was dose-dependently decreased in the Pio. and LGP1-treated groups by 36.59%, 22.99%, 24.54%, and 32.71%, respectively. LGP2 treatment had no hypoglycaemic effect in any group .

**Figure 3:**
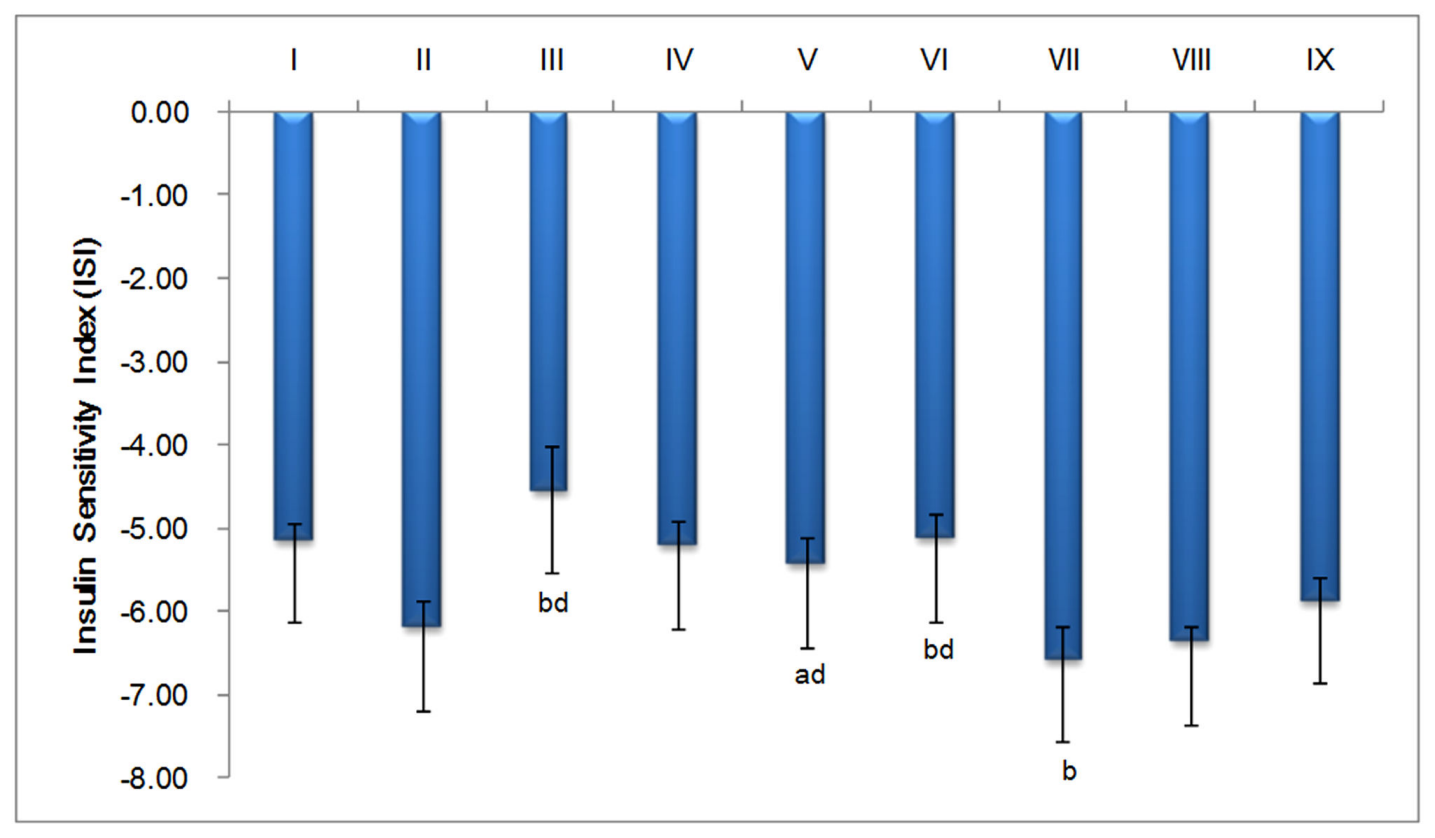
Effect of LGP1 and LGP2 on insulin sensitivity index (ISI). i: Normal control; ii: Model control; iii: 10 mg.kg^-1^.d^-1^ of Pioglitazone; iv-vi: 20, 40, 80 mg.kg^-1^.d^-1^ of LGP1; vii-ix: 20, 40, 80 mg.kg^-1^.d^-1^ of LGP2. The results were presented as the means ±SD. ***^a^*** *P*<0.05 and ^b^ *P*<0.01 as compared with normal control group; ^c^ *P*<0.05 and ^d^ *P*<0.01 as compared with model group.

FINS levels in the normal group (21.99±3.24 μIU/ml) were significantly higher than the Pio.-treated group (12.85±5.88 μIU/ml, *p*<0.05), the LGP1 medium dose-treated group (18.64±3.59 μIU/ml, *p*<0.05), and the high dose-treated group (16.83±2.25 μIU/ml, *p*<0.01) (Figure **2b**). The ISI in the Pio. and LGP1 low dose-treated groups was significantly decreased (*p*<0.01) compared with the normal group. FINS levels were significantly higher (*p*<0.01) in the model control mice (25.48±5.44 μIU/ml) than that in Pio.or the LGP1 medium and high dose–treated groups. The Pio. and LGP1-treated group ISIs were significantly higher (*p*<0.01) than the model group (Figure **3**).

For LGP2 having no hypoglycemic effect under the dosage of no more than 80 mg.kg^-1^.d^-1^, therefore, we didn’t do the H&E staining, apoptosis of cells, and the expression of caspase-3, 8, 9, NF-κB, Bax, Bcl-2 test in any group for further mechanism analysis.

### 3.2: Histopathological findings

**Figure 4:**
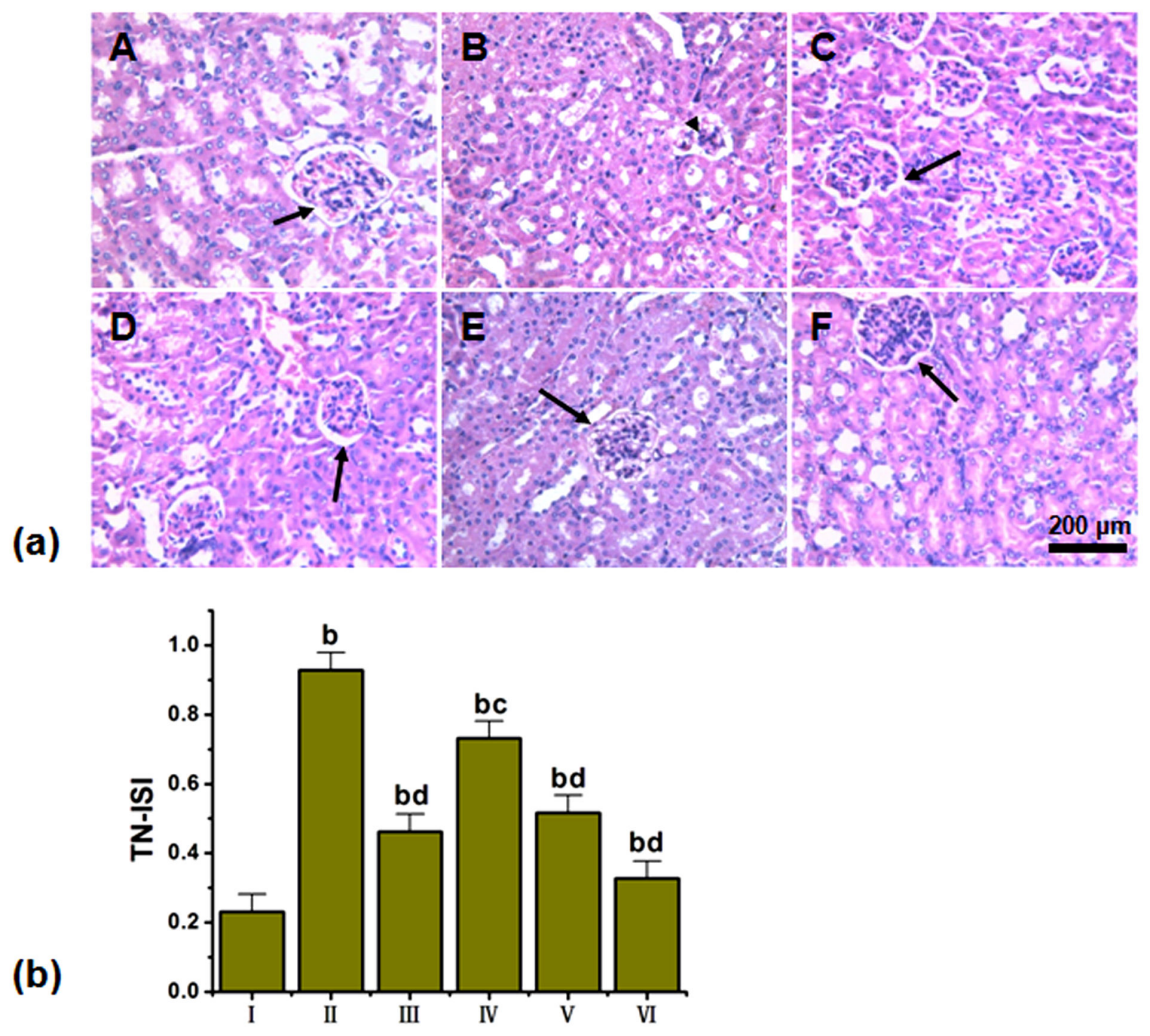
(a) LGP1 treatment alleviated pathological injuries in mouse kidney (HE staining, scale bar: 200 μm). (A) Normal control; (B) Model control; (C) 10mg.kg^-1^.d^-1^ of Pioglitazone; (D) 20mg.kg^-1^.d^-1^ of LGP1; (E) 40mg.kg^-1^.d^-1^ of LGP1; (F) 80mg.kg^-1^.d^-1^ of LGP1. Arrows represented the glomerulus. Triangle represented the damaged glomerulus. (**b**) Kidney damages were assessed and represented by the tubular necrosis-individual severity index (TN-ISI) score. Results were presented as the means ±SD. ***^b^*** *P*<0.01 as compared with normal control group; ^c^ *P*<0.05 and ^d^ *P*<0.01 as compared with model group.

H&E staining of the STZ-induced mouse kidneys revealed renal tubule hypertrophy, vacuolar degeneration, slight inflammatory cell infiltration, and apparent glomerular collapse. After the 14-day LGP1 treatment, the tubule hypertrophy, vacuolar degeneration and inflammatory cell infiltration in the STZ-induced mice were significantly improved. The LGP1 control group exhibited minimal variation in these histological changes compared with normal controls. Concomitantly, the histological studies of high dose-treated animal kidneys revealed less renal tubule expansion and glomerular basement membrane thickening compared with the STZ-induced mice (Figure **4a-b**).

### 3.3 Cell Apoptosis

**Figure 5:**
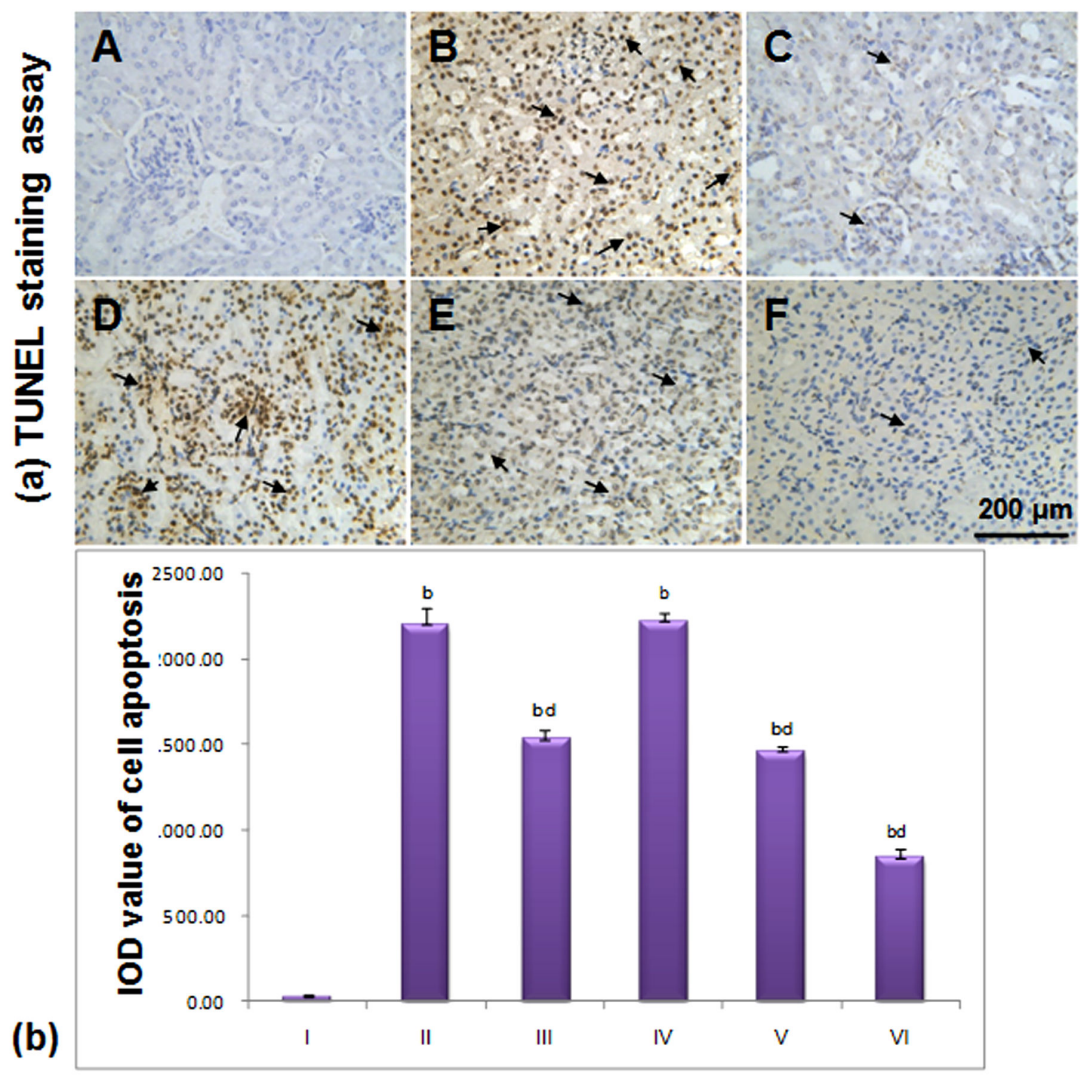
(a) LGP1 treatment inhibited the apoptosis in mouse kidney tissue (TUNEL staining assay, scale bar: 200 μm). (A) Normal control; (B) Model control; (C) 10mg.kg^-1^.d^-1^ of Pioglitazone; (D) 20mg.kg^-1^.d^-1^ of LGP1; (E) 40mg.kg^-1^.d^-1^ of LGP1; (F) 80mg.kg^-1^.d^-1^ of LGP1. Arrows represented the apoptotic cell. (**b**) The IOD value of positive expression for cell apoptosis. Results were presented as the means ±SD. ***^a^*** *P*<0.05 and ^b^ *P*<0.01 as compared with normal control group; ^c^ *P*<0.05 and ^d^ *P*<0.01 as compared with model group.

After TUNEL and haematoxylin staining, apoptotic cell nuclei stained brown while normal nuclei stained blue. There were few apoptotic cells, which revealed a significant difference (*p*<0.01) in the normal group compared with the model, Pio. and LGP1-treated groups. In the model group, apoptosis was significantly increased (*p*<0.01) compared with the Pio. and LGP1-treated groups (40, 80 mg. kg^-1^.d^-1^) as shown in Figure **5a**. After LGP1 treatment for 14 days, apoptotic cells were significantly decreased in a dose-dependent manner (Figure **5b**).

### 3.4: Caspase-3, 8, 9 activities

**Figure 6:**
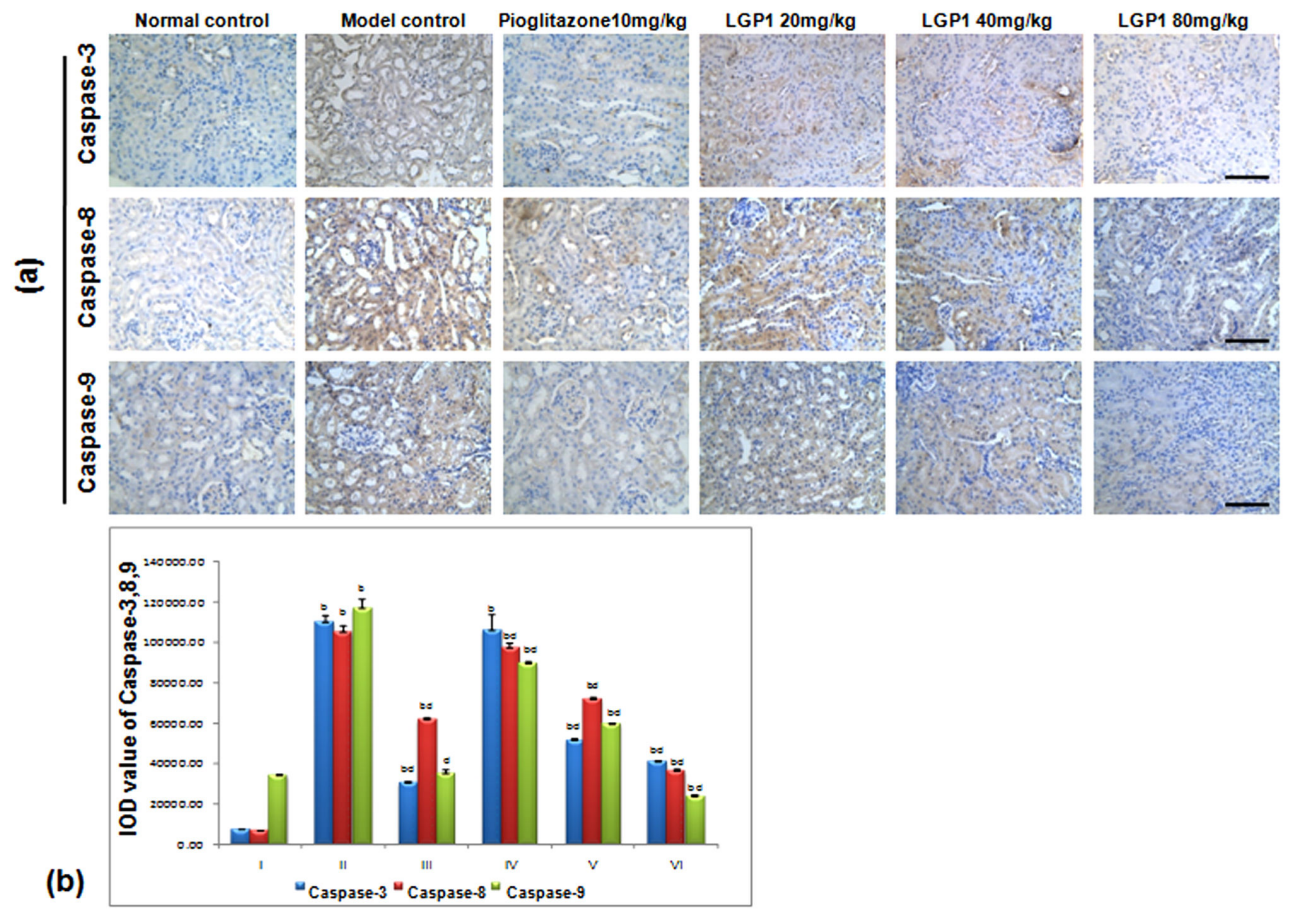
(a) LGP1 treatment reduced the expressions of Caspase-3, 8, 9 in mouse kidney tissue (Immunohistochemical analysis, scale bar: 200 μm). (A) Normal control; (B) Model control; (C) 10mg.kg^-1^.d^-1^ of Pioglitazone; (D) 20mg.kg^-1^.d^-1^ of LGP1; (E) 40mg.kg^-1^.d^-1^ of LGP1; (F) 80mg.kg^-1^.d^-1^ of LGP1. (**b**) The IOD value of positvie expression for Caspase-3, 8, 9. Results were presented as the means ±SD. ***^a^*** *P*<0.05 and ^b^ *P*<0.01 as compared with normal control group; ^c^ *P*<0.05 and ^d^ *P*<0.01 as compared with model group.

 In the normal mice, caspase-3, 8, 9 protein expression was present in the cytoplasm (Figure **6a**), which was significantly different compared with the treated animals (*p*<0.01). Caspase-3, -8, -9 expression increased in the STZ-induced mice, and the caspase-3 IOD value significantly increased compared with the Pio. and LGP1 40, 80 mg.kg^-1^.d^-1^-treated groups (*p*<0.01). The caspase-8 and -9 IOD values both increased significantly compared with Pio. and LGP1-treated groups (*p*<0.01) (Figure **6b**).

### 3.5 NF-κB

**Figure 7:**
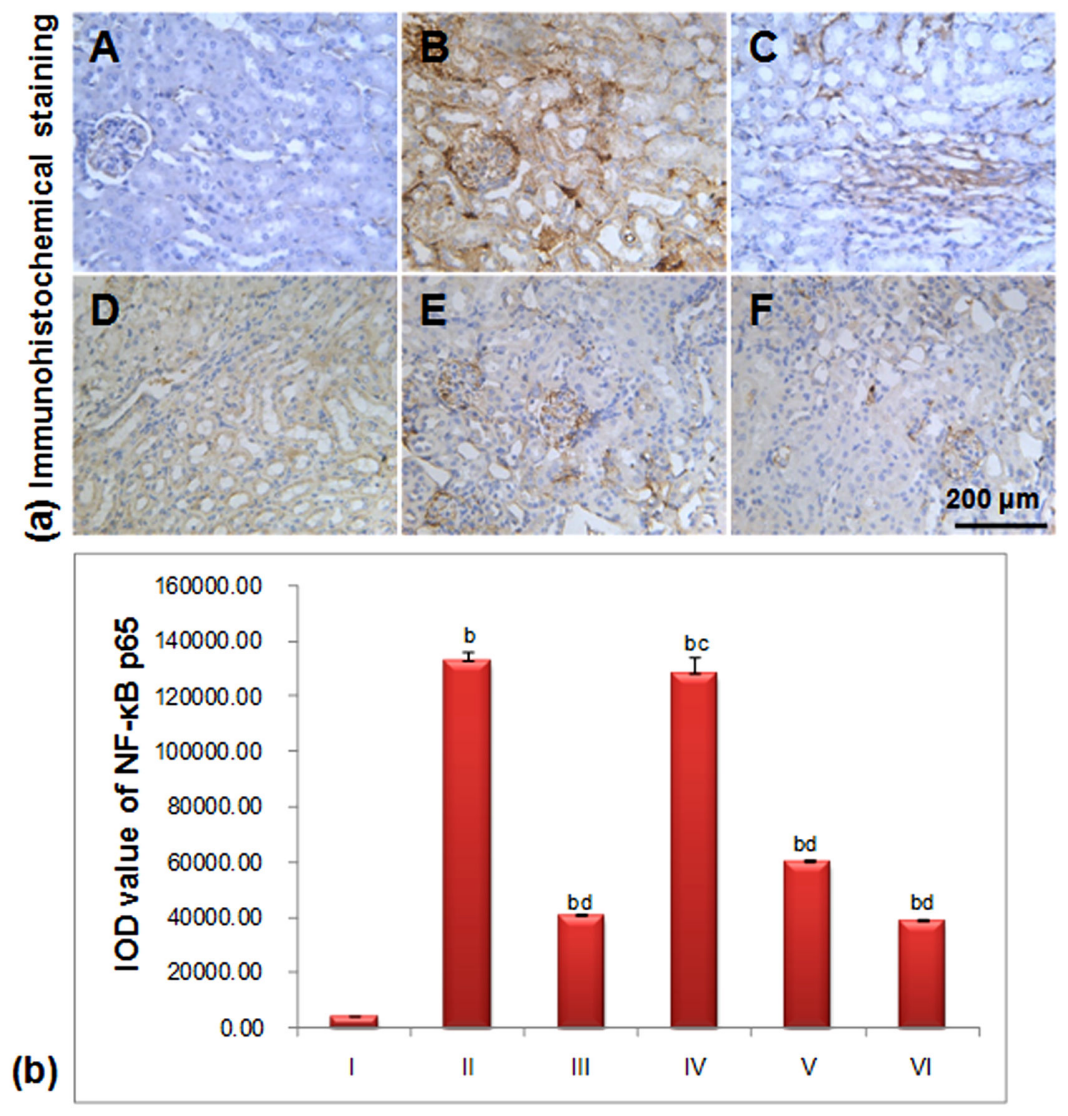
(a) LGP1 decreased the NF-κB p65 expression in mouse kidney tissue (Immunohistochemical analysis, scale bar: 200 μm). (A) Normal control; (B) Model control; (C) 10mg.kg^-1^.d^-1^ of Pioglitazone; (D) 20mg.kg^-1^.d^-1^ of LGP1; (E) 40mg.kg^-1^.d^-1^ of LGP1; (F) 80mg.kg^-1^.d^-1^ of LGP1. Arrows represented the apoptotic cell. (**b**) The IOD value of positive expression for NF-κB p65. Results were presented as the means ±SD. ***^a^*** *P*<0.05 and ^b^ *P*<0.01 as compared with normal control group; ^c^ *P*<0.05 and ^d^ *P*<0.01 as compared with model group.

In the normal group, there was only slight NF-κB-positive brown staining in the glomerulus, renal interstitium and tubule, and its IOD value was significantly different compared with the treated animals (*p*<0.01) as shown in Figure **7b**. Conversely, in the model group, the positive staining in the glomerulus, renal interstitium and tubules was deeper than the normal mice, which implied that NF-κB-positive staining as well as apoptotic cell number increased (Figure **7a**). The STZ-induced mouse IOD value was significantly different than the 40, 80 mg.kg^-1^.d^-1^ LGP1-treated groups (*p*<0.01) and the 20 mg.kg^-1^.d^-1^ group (*p*<0.05).

After LGP1 treatment for 14 days, the NF-κB positive staining in the glomerulus, renal interstitium and tubule was slightly less brown than that in the model group. The NF-κB expression was obviously decreased in the high dose group (Figure **7a**).

### 3.6 Bax/Bcl-2

Apoptosis-promoting factor Bax and anti-apoptotic factor Bcl-2 are Bcl-2 family members that play an important role in inhibiting cell apoptosis and extending cell life. Bax and Bcl-2 form a dimer that regulates protein and nucleic acid enzyme activities and cell apoptosis in two ways. The Bax/Bcl-2 ratio determines whether cells undergo apoptosis. Bcl-2 protein over-expression causes dimerisation with all of the cellular Bax protein to inhibit apoptosis. Conversely, when Bax protein was over-expressed, it combined with Bcl-2 and formed a heterodimer that promoted apoptosis[18,29].

**Figure 8:**
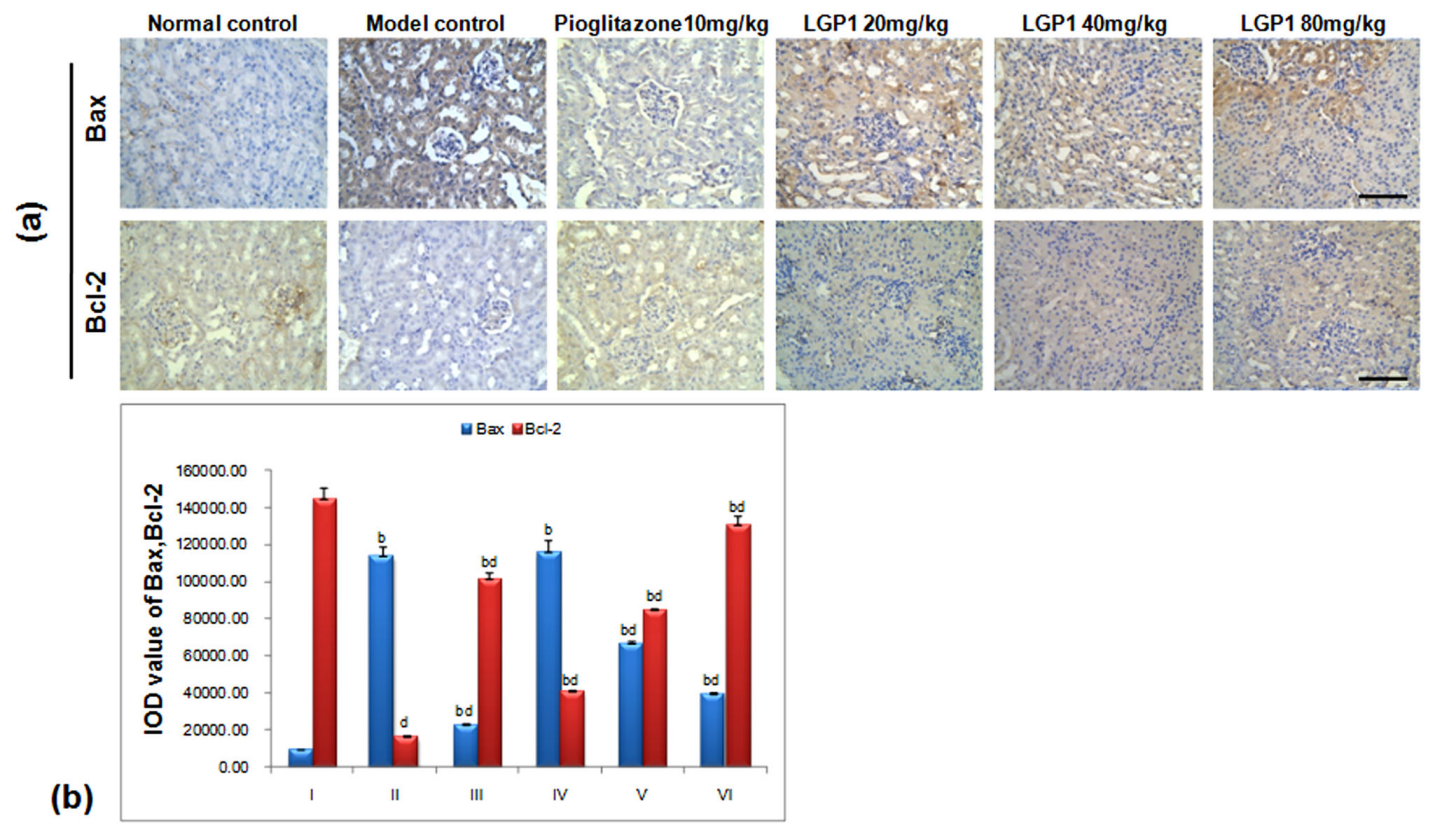
(a) LGP1 down-regulated the Bax expression and up-regulated the Bcl-2 level in mouse kidney tissue (Immunohistochemical analysis, scale bar: 200 μm). (A) Normal control; (B) Model control; (C) 10mg.kg^-1^.d^-1^ of Pioglitazone; (D) 20mg.kg^-1^.d^-1^ of LGP1; (E) 40mg.kg^-1^.d^-1^ of LGP1; (F) 80mg.kg^-1^.d^-1^ of LGP1. Arrows represented the apoptotic cell. (**b**) The IOD value of positive expression for Bax and Bcl-2. Results were presented as the means ±SD. ***^a^*** *P*<0.05 and ^b^ *P*<0.01 as compared with normal control group; ^c^ *P*<0.05 and ^d^ *P*<0.01 as compared with model group.

In the normal mice, there were only had a few cells with cytoplasmic Bax staining. However, Bax-positive staining was obvious in the STZ-induced mice, and its expression in the normal and model groups were significantly different compared with the LGP1-treated groups (*p*<0.01) as seen in Figure **8**. After LGP1 treatment (20, 40, 80 mg. kg^-1^.d^-1^) for 14 days, Bax expression in each group was obviously reduced. Bcl-2 cytoplasmic expression was obvious in the normal mice. Conversely, there were only a few Bcl-2 positive cells in the model mice. Bcl-2 expression in the normal, model groups were significantly different compared with each LGP1-treated group (*p*<0.01). After LGP1 treatment (20, 40, 80 mg. kg^-1^.d^-1^) for 14 days, Bcl-2 protein expression was obviously induced in each group (Figure **8a-b**).

## Discussion

 Increasing amounts of literature have demonstrated that β-cell apoptosis is not only the direct cause of type 1 diabetes, but is also an important factor in T2DM pathogenesis[30,31]. Hyperglycaemia, FFAs, NO, proinflammatory cytokines and reactive oxygen species induce β-cell inflammation, which promotes their programmed death[32,33]. Caspases are the “central processing units” that mediate β-cell apoptosis and are involved in DM occurrence and development[15,17]. NF-κB plays a critical role in inflammation and T2DM development. It was widely expressed in the cells and regulated inflammatory gene transcription and inflammation as well as apoptosis[34]. The immunohistochemistry results revealed that LGP1 treatment significantly reduced NF-κB expression and inhibited its activation, which probably is related to IκB degradation. IκB was phosphorylated by the I-kappa B kinase complex (IKK), and Lys21 and 22 were ubiquitinated within the molecule. Finally, IκB degradation exposed the NF-κB nuclear localization sequence to cause gene transcription, which inhibited β-cell apoptosis induced by inflammatory factors such as TNF-α and IL-1β and improved IR[8].

Diabetic nephropathy (DN) is the most common microvascular complication and is a leading cause of death in T2DM patients. Its early symptoms often include glomerular hypertrophy, excessive extracellular matrix amassing, and glomerular and tubular basement membrane thickening[35]. In this research, we determined that LGP1 treatment promoted hypoglycaemia in the STZ-induced diabetic mice, and the histopathological examination also revealed that LGP1 treatment reduced swelling, inflammation, cell infiltration and renal tubular vacuolar degeneration.

The caspase family plays a key role in apoptosis. Once the caspase cascade reaction is activated it cannot be reversed, which resulted in apoptosis. There are 4 pathways that are associated with apoptotic signal transduction (1). In mitochondria- dependent apoptosis caspase-9 is activated when conjugated with the apoptotic protease- activating factor 1, which triggers pancreatic islet β-cell apoptosis (2). In the death receptor pathway, the death receptor ligand system Fas/FasL activates caspase-8, -3 and induces cell apoptosis[36]. (3) In endoplasmic reticulum stress, calcium-dependent protein kinases are activated by calcium-ion balance disorders, and they directly cut and activate caspase-12, which promotes caspase-7 and Bim translocation from the cytoplasm to the endoplasmic reticulum surface, which activated caspase-12 to further promote the cascade[37]. (4) In the granule enzyme B pathway, caspase-3 is activated via granule enzyme B cleavage, which changes bid into tBid. Caspase activation cleaves the apoptosis inhibitor protein and DNA repairing enzymes to produce DNA fragments, resulting in β-cell apoptosis. In STZ-induced diabetic mice, the caspase family including caspase-3, 8, 9 played a major role in pancreatic islet β-cells apoptosis. Caspase-6, 7 and the death degradation substrate were triggered via caspase-3 activation. As the crucial initiator caspase family member, caspase-8 activation was the first step in the cascade[38].

Our experimental results demonstrate that LGP1 treatment significantly reduced caspase-3, -8, -9 expression and inhibited renal cell apoptosis. This mechanism would be associated with the following pharmacological pathways: (1) LGP1 combined with 3 membrane FAS molecules and bonded with the cytoplasmic Fas-associated death domain (FADD) via the death domain (DD). LGP1 then combined with the death effector domain (DED) of the nearby caspase-8 via the DED of FADD, which formed caspase-8 oligosaccharides to promote self-cleaving and activation and activated the downstream caspase-3 and induced cell death (2). After TNF and TNFR-1 conjunction, three TNFR-1 dimers combined with TNFR-associated death domain (TRADD) in the tumour necrosis factor receptor protein via the DD domain. The TRADD DD domain combined with FADD, downloaded an apoptosis signal, activated caspase-8, and then activated the downstream caspase-3, which caused apoptosis.

LGP1 was isolated for the first time from ACLR. Though the hypoglycaemic effect of LGP1 was not better than Pio. treatment, it could prevent or delay T2DM development via decreasing apoptotic cells, thus increasing the ISI and improving IR. Our results also demonstrated that LGP1 treatment significantly reduced FBG and FINS levels in the STZ-induced diabetic mice. This mechanism may be associated with increasing ISI and improving IR via inhibiting the cysteine protease caspase family and including caspase-3, -8, -9, which are involved in apoptosis.
